# From Caries to Periodontal Breakdown: A Biological and Clinical Continuum Linking Cariology, Operative Dentistry, Endodontics, and Periodontology

**DOI:** 10.3390/dj14060380

**Published:** 2026-06-18

**Authors:** Yasir Dilshad Siddiqui, Nusrat Sultana, Osama Khattak, Mohammed Zahedul Islam Nizami

**Affiliations:** 1 Department of Preventive Dentistry, College of Dentistry, Jouf University, Sakaka 72345, Saudi Arabia; ydsiddiqui@ju.edu.sa; 2 Graduate School of Natural Science and Technology, Okayama University, 3-1-1 Tsushimanaka, Kita-Ku, Okayama 700-8530, Japan; zahidnusrat16@gmail.com; 3 Department of Restorative Dentistry, College of Dentistry, Jouf University, Sakaka 72345, Saudi Arabia; okhattak@ju.edu.sa; 4 Mineralized Tissue Biology, ADA Forsyth Institute, 100 Chestnut Street, Somerville, MA 02143, USA

**Keywords:** clinical continuum, cariology, operative dentistry, endodontics, periodontology

## Abstract

Dental diseases have long been taught and treated as separate entities: cariology, operative dentistry, endodontics, and periodontology, each working within its own boundaries. However, increasing biological and clinical evidence suggests that this classified view does not fully reflect how disease progresses in the mouth. Instead, dental disease should be understood as a continuum within the interconnected tooth–pulp–periodontium complex. This review provides current evidence showing how dental caries can serve as the starting point of a process that can progress through pulpitis and apical periodontitis and eventually affect surrounding periodontal tissues. Caries is now widely known as a biofilm-driven and host-influenced condition shaped by ecological imbalance rather than specific pathogens alone. As lesions penetrate deeper into dentin, the structure becomes more permeable, permitting diffusion of microbial metabolites and signaling molecules toward the pulp. This initiates a multifaceted inflammatory reaction within the pulp tissue. At this stage, pulpitis becomes a critical turning point, where the outcome depends on microbial load, lesion activity, host response, and quality of clinical intervention. If the disease is not well controlled, it may lead to pulp necrosis, allowing infection to spread beyond the root canal and initiate periapical inflammation. Through anatomical pathways such as apical foramina and lateral canals, these processes can extend further, sometimes resembling or overlapping with periodontal disease. This overlap creates diagnostic challenges, as conventional tests may not always distinguish between conditions. A structured, pathway-based diagnostic approach is therefore essential. From a treatment perspective, this continuum model highlights early intervention, minimally invasive care, preservation of pulp vitality when possible, and maintenance of a strong coronal seal. Ultimately, stronger integration across dental disciplines can improve diagnosis, guide treatment decisions, support long-term tooth preservation, and promote unified dental education. This article presents a narrative review supported by a structured literature search and proposes a clinically actionable framework that extends established endodontic–periodontal concepts upstream to include caries initiation and restorative modulation.

## 1. Introduction

Dental education has traditionally been structured into separate clinical disciplines, including cariology and operative dentistry, endodontics, and periodontology, each of which is defined by anatomical boundaries and a specific clinical scope. This discipline-based structure facilitates procedural specialization and streamlines curriculum design, but it does not fully reflect how dental disease manifests in real patients. In clinical practice, conditions affecting enamel, dentin, pulp, and periodontium rarely occur in isolation and instead progress along shared biological pathways that cross traditional disciplinary boundaries [1,2,3,4]. Despite major advances in prevention and treatment, dental caries remains the most prevalent chronic disease worldwide. Recent estimates from the 2021 Global Burden of Disease study suggest that approximately 2.37 billion people are affected by caries in permanent teeth, making untreated caries the single most common health condition globally [5,6]. As caries progresses, pulpitis and apical periodontitis often arise as subsequent stages in disease progression, whereas severe periodontitis affects nearly 12.5% of adults worldwide and remains a leading cause of tooth loss [5,7,8,9]. Although these conditions are closely linked in their initiation and progression, they are still frequently managed as separate clinical entities.

Growing biological, histopathological, and microbiological evidence challenges long-standing compartmentalized models that treat cariology, endodontics, and periodontology as separate disease entities. Rather than separate processes, recent research highlights a tooth–pulp–periodontium continuum in which caries-associated biofilm imbalance initiates a host-driven inflammatory response sequence. Cariogenic biofilms promote demineralization of enamel and dentin, facilitating the transport of bacterial metabolites, including lipopolysaccharides and proteolytic enzymes, through the dentinal tubules. This process triggers innate and adaptive immune responses within the pulp, characterized by infiltration of immune cells such as macrophages and lymphocytes, along with increased cytokine activity [10,11,12]. As pulpal inflammation progresses, microbial components and inflammatory mediators can extend beyond the pulp chamber through the apical foramina, lateral and accessory canals, and cemental discontinuities. This spread creates direct biological interactions with periapical and periodontal tissues [13,14]. Experimental models have demonstrated that pulpal and periapical infections upregulate interleukin-1β, tumor necrosis factor-α, chemokines, and matrix metalloproteinases. These mediators stimulate osteoclast activation, resulting in bone loss and breakdown of periodontal attachment [12,15,16,17]. Clinically, histologic and microbiologic studies have shown that teeth with deep caries frequently present with concurrent pulpal necrosis and apical inflammation. These findings, along with associated localized periodontal destruction, reinforce that these conditions are interconnected manifestations of a shared pathogenic process instead of independent disease entities [18,19,20].

The lack of recognition of this biological continuum contributes to diagnostic ambiguity, inappropriate sequencing of care, and unnecessarily aggressive interventions. For example, periodontal probing defects of endodontic origin may be misinterpreted as primary periodontal disease. Likewise, deep carious lesions associated with subclinical pulpal inflammation are sometimes restored without sufficient biological consideration, predisposing the tooth to delayed pulp necrosis and subsequent apical disease. An integrated disease model is therefore critical for accurate diagnosis, realistic prognosis, minimally invasive treatment planning, and long-term tooth preservation [13,21,22].

**Figure 1:**
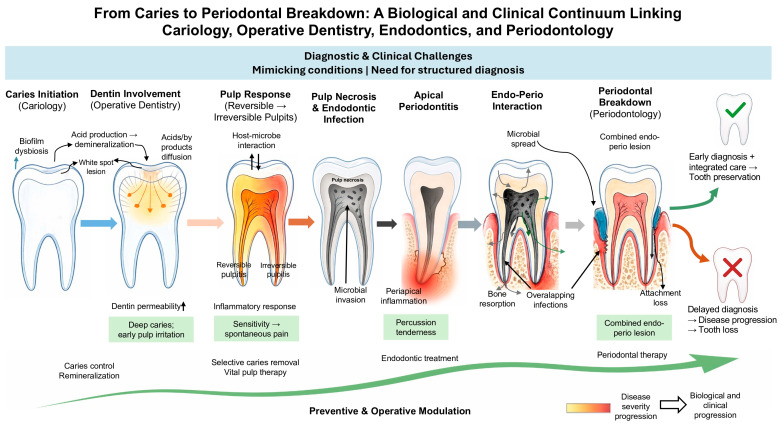
This schematic illustrates the biological and clinical continuum linking cariology, operative dentistry, endodontics, and periodontology within the interconnected tooth–pulp–periodontium complex. Dental caries begin with biofilm dysbiosis and acid-mediated enamel demineralization, then progress into dentin, where increased permeability allows diffusion of bacterial byproducts and inflammatory mediators. This stimulates a host-mediated pulpal response ranging from reversible to irreversible pulpitis, a critical transition point in disease progression. If uncontrolled, persistent inflammation may lead to pulp necrosis and microbial colonization of the root canal system, followed by spread of infection into the periapical tissues, causing apical periodontitis and bone resorption. Through anatomical pathways such as the apical foramen, lateral canals, and dentinal tubules, infection may extend between the endodontic and periodontal compartments, producing overlapping endodontic–periodontal lesions and, in susceptible cases, periodontal breakdown. The upper band highlights major diagnostic and clinical challenges, including overlapping presentations and the need for a systematic, biologically guided diagnostic framework. The lower pathway emphasizes prevention and treatment, including caries control, remineralization, selective caries removal, vital pulp therapy, endodontic care, and periodontal therapy. The final panel shows that early diagnosis and integrated interdisciplinary care favor tooth preservation, whereas delayed or fragmented management can lead to progression and tooth loss.

The objective of this paper is to integrate current biological, microbiological, and clinical evidence supporting an integrated view of caries, pulpitis, apical periodontitis, and periodontal breakdown as biologically linked stages within a disease continuum. Special focus is given to cariology as the initiating event and pulpitis as a central biological crossroads connecting restorative, endodontic, and periodontal outcomes. This continuum framework and its diagnostic/therapeutic implications are summarized in Figure 1.

This review contributes by integrating dentin-mediated pulpal/apical and periodontal processes into one connected biological and clinical pathway. Unlike prior reviews that often address these conditions separately or focus mainly on endo–perio lesions, this review traces disease progression from caries-driven biofilm dysbiosis to pulpal inflammation, pulp necrosis, intraradicular infection, periradicular inflammation, and periodontal-like breakdown through anatomical communication routes. It also provides clinically relevant frameworks explaining why endodontic and periodontal diseases may appear similar and cause diagnostic errors. The review emphasizes structured differential diagnosis, staged treatment sequencing, and endodontic-first management when an intraradicular source is suspected. Finally, it highlights upstream interventions, including caries control, selective caries removal, vital pulp therapy, and coronal sealing, as key determinants of long-term tooth preservation.

### 1.1. Review Methodology

This article was designed as a narrative review supported by a structured literature search. Its aim was to synthesize biological, microbiological, histopathological, and clinical evidence linking dental caries, operative dentistry, pulpal inflammation, apical periodontitis, and periodontal breakdown within an integrated tooth–pulp–periodontium continuum. The review was not intended as a systematic review or meta-analysis; therefore, protocol registration, formal risk-of-bias assessment, and quantitative evidence synthesis were not performed.

A structured search was conducted using PubMed/MEDLINE, Scopus, Web of Science, and Google Scholar. The search focused primarily on literature published between 2000 and 2026, although earlier landmark studies were included when they provided foundational concepts related to caries progression, pulp–periodontium communication, endodontic–periodontal lesions, or minimally invasive caries and pulp management. Search terms used alone and in combination included dental caries, biofilm dysbiosis, dentin permeability, pulpitis, vital pulp therapy, pulp necrosis, apical periodontitis, endodontic infection, endo–perio lesion, periodontal breakdown, pulp–periodontium communication, lateral canals, accessory canals, coronal leakage, selective caries removal, and tooth preservation.

Eligible sources included clinical studies, in vitro and ex vivo investigations, animal and histological studies, systematic and narrative reviews, consensus reports, position papers, and clinical guidelines relevant to cariology, operative dentistry, endodontics, and periodontology. Studies were included if they addressed caries-related biofilm dysbiosis, dentin as a biological interface, pulpal inflammatory responses, progression to necrosis and apical periodontitis, anatomical pulp–periodontium communication, endodontic–periodontal lesion classification, diagnostic challenges, or interdisciplinary treatment sequencing. Articles were excluded if they were unrelated to dental disease progression, lacked relevance to the tooth–pulp–periodontium complex, focused only on restorative materials without biological or clinical linkage, or were unavailable in English. Case reports were considered only when they illustrated clinically relevant diagnostic overlap and were not used as primary evidence for general conclusions.

The selected literature was evaluated qualitatively, with priority given to systematic reviews, consensus statements, clinical guidelines, and well-designed clinical or experimental studies. Evidence was synthesized thematically according to the proposed pathway: caries initiation, dentin involvement, pulpal inflammation, pulpal necrosis, apical periodontitis, endodontic–periodontal communication, periodontal-like breakdown, diagnosis, and treatment sequencing.

### 1.2. Novelty and Contribution of the Continuum Model

Pulp–periodontium communication through the apical foramen, lateral and accessory canals, furcation canals, dentinal tubules, cemental defects, perforations, and fractures is well established. Thus, this review does not present these anatomical or biological relationships as new. Rather, its novelty lies in reframing them within a broader caries-initiated and clinically actionable continuum.

Traditional endodontic–periodontal models often emphasize communication between infected root canal systems and periodontal tissues after pulpal necrosis or established endodontic disease. In contrast, this framework begins earlier by positioning cariology and operative dentistry as upstream determinants of pulpal, periapical, and periodontal outcomes. By integrating caries biofilm dysbiosis, dentin permeability, pulpal inflammation, restorative sealing, endodontic infection, and periodontal-like breakdown into a single pathway-based model, this review provides a practical structure for diagnosis, treatment sequencing, and tooth-preserving interdisciplinary care.

This continuum model does not replace existing endodontic–periodontal classifications. Instead, it complements them by emphasizing that many endodontic–periodontal presentations may originate from earlier cariologic and restorative events. This perspective highlights opportunities for earlier intervention through caries control, selective caries removal, vital pulp therapy, durable coronal sealing, and structured reassessment before advanced endodontic or periodontal breakdown occurs.

## 2. Cariology: Disease Starting Point

### 2.1. Dental Caries—Biofilm-Mediated Host-Modulated Disease

Dental caries is now widely understood as a chronic, noncommunicable disease driven by biofilm dysbiosis rather than a classic infection caused by individual pathogens. Evidence shows that environmental pressures, particularly frequent sugar intake, disrupt the normal symbiosis of the dental biofilm and promote disease-associated community shifts rather than infection by single organisms [23,24]. The ecological plaque hypothesis offers a widely accepted biological explanation for how caries begins and progresses over time. It describes disease as the result of environmentally driven changes in the composition and metabolic activity of the dental biofilm rather than the action of specific pathogens alone [25,26]. According to this model, the normally diverse and balanced oral microbiota shifts toward dominance by acidogenic and acid-tolerant microorganisms when repeatedly exposed to fermentable carbohydrates, especially sucrose. These factors drive sustained acid production at the tooth–biofilm interface and selectively favor cariogenic communities [27,28].

Repeated carbohydrate exposure leads to prolonged biofilm acidification, disrupting mineral homeostasis by tipping the balance toward net demineralization of enamel and dentin. Over time, this persistent metabolic imbalance results in the clinical expression of carious lesions [29,30]. By integrating microbial, biochemical, and ecological principles, the ecological plaque hypothesis explains why sugar frequency, pH fluctuations, and community-level metabolic shifts are the primary drivers of caries, framing it as a chronic disease of biofilm imbalance rather than a traditional infection by a single pathogen [23,31].

Importantly, contemporary molecular studies have demonstrated that caries-associated biofilms are functionally dysbiotic rather than pathogen-specific. These communities consist of complex microbial consortia, including *Lactobacillus*, *Scardovia*, *Bifidobacterium*, and *Nonmutans streptococci*, which collectively maintain low-pH microenvironments through shared metabolic activity. This highlights that metabolic output, rather than microbial presence alone, drives enamel and dentin demineralization [32,33,34,35].

Caries progression reflects a dynamic imbalance between demineralization and remineralization, influenced by host-related natural protective factors such as salivary flow and buffering capacity, calcium–phosphate availability, fluoride exposure, and immune components, including immunoglobulins and antimicrobial peptides [36,37,38]. As a result, lesion behavior is highly heterogeneous: some lesions progress rapidly toward the pulp, whereas others remain arrested for long periods, even in the presence of similar microbial profiles.

From a biological perspective, caries should therefore be viewed as a chronic inflammatory disease of the tooth surface, with consequences that extend beyond mineral loss. As lesions advance into dentin, the process shifts from a surface-confined phenomenon to a biologically active interface capable of influencing pulpal health and, ultimately, periapical and periodontal tissues.

### 2.2. Dentin: Biological Interface Between Caries and the Pulp

Once caries penetrates enamel, dentin becomes the key determinant of disease progression. Dentin is not an inert substrate but a biologically active, responsive tissue with a tubular microarchitecture that directly links the external environment to the dental pulp. As lesions deepen, the increasing diameter and density of dentinal tubules markedly increase permeability, allowing for more rapid molecular diffusion toward the pulp [39,40,41].

This tubular network allows microbial metabolites, including organic acids, lipopolysaccharides, proteases, and other virulence factors, to diffuse toward the pulp well before bacteria physically invade pulpal tissue. Experimental and histologic studies consistently show inflammatory changes in the pulp beneath carious dentin even when bacteria are not detectable within the pulp chamber, underscoring that toxin diffusion, rather than direct bacterial invasion, is often the primary driver of early pulpitis [14,18,42].

In addition to serving as a passive conduit, dentin actively participates in host defense. Odontoblasts express pattern-recognition receptors, including Toll-like receptors, and respond to bacterial byproducts by releasing cytokines, chemokines, and antimicrobial peptides, effectively functioning as immune-competent cells [43,44]. While these responses are intended to limit microbial challenge, they also contribute to local inflammation and neurovascular changes within the pulp.

The biological impact of dentin permeability is greater in teeth with exposed root surfaces, cervical lesions, noncarious cervical defects, or developmental enamel defects, where dentin may contact the oral environment directly. In these situations, even mild microbial imbalance can trigger pulpal inflammation, reinforcing the critical role of cariology on coronal and root surfaces as a precursor to pulpal disease.

### 2.3. Pulpal Defense Mechanisms Triggered by Caries

The dental pulp responds to caries-related injury in a graded and adaptive way, depending on how intense, long-lasting, and close the lesion is to the pulp. In early or slowly progressing lesions, surviving odontoblasts can lay down reactionary dentin, forming a protective barrier that reduces dentin permeability and limits toxin diffusion [45,46]. If the cariogenic challenge is brought under control, this protective response can be enough to preserve pulpal health. In contrast, rapidly advancing or deep carious lesions often lead to odontoblast death, which triggers reparative dentin formation by pulp progenitor or stem cells. Although reparative dentin creates a structural barrier, it is usually irregular and poorly organized, reflecting the biological cost of tissue injury. Moreover, inflammatory mediators such as prostaglandins, neuropeptides, and cytokines are released, altering pulpal blood flow, sensory nerve activity, and vascular permeability [47,48].

Crucially, histologic evidence shows that pulpal inflammation is frequently spatially heterogeneous, with focal areas of intense inflammation adjacent to relatively preserved tissue [13]. This challenges the traditional binary classification of pulpitis as “reversible” or “irreversible” and instead supports a continuum-based model in which pulpal status cannot be reliably inferred from clinical symptoms alone. Once inflammation becomes persistent or exceeds the ability of the tissue to repair itself, the pulp becomes increasingly vulnerable to operative trauma, bacterial leakage, and eventual necrosis.

### 2.4. Operative Interventions as Biological Modulators of Caries–Pulp Interactions

Operative dentistry serves as a key transition stage along the caries–pulp continuum. Historically, nonselective caries removal aimed at eliminating all softened dentin, but this approach often led to unnecessary pulp exposure and irreversible pulpal injury [49]. In contrast, contemporary evidence strongly supports selective and stepwise caries removal, as preserving affected dentin can help maintain pulpal vitality while still substantially reducing the bacterial burden [50,51,52].

Moreover, restorative treatment itself represents a biological challenge to the pulp. Thermal stress, dentin dehydration, mechanical vibration, and polymerization shrinkage can worsen pulpal inflammation, especially in teeth already compromised by caries-related changes [53,54]. Even without direct pulp exposure, these stressors may push the tissue from reversible inflammation toward irreversible inflammation. Microleakage is a major concern because it allows bacterial byproducts to continuously diffuse beneath restorations. Ex vivo studies in extracted human teeth have shown that microorganisms can persist at and penetrate the cavity–restoration interface long after placement, directly linking marginal gaps with bacterial colonization [55,56].

Complementary in vivo histologic evidence shows that poorly sealed restorations can trigger pronounced pulpal inflammation and tissue damage, even when caries removal initially appears clinically successful. Notably, compared with conventional composites, compomer restorations cause less severe pulpal inflammation in animal models, likely reflecting differences in material biocompatibility and interfacial sealing [57,58]. Longitudinal in vivo data further confirm that the extent of bacterial microleakage directly correlates with the magnitude of pulpal tissue responses across restorative materials [54]. Taken together, these findings indicate that restoration-driven pulpitis represents a biologically distinct, yet clinically common, pathway linking cariology and endodontics.

Collectively, these observations highlight that operative dentistry is more than just mechanical defect repair; it is a biological intervention with long-term consequences. The quality of caries management and restorative sealing directly influences pulpal survival and, ultimately, downstream periapical and periodontal health.

### 2.5. Cariology as the Foundation for Endodontic and Periodontal Sequelae

Deep carious lesions that do not resolve or are inadequately managed can eventually lead to pulp necrosis, turning the pulp chamber into a reservoir for anaerobic biofilms. Once necrosis develops, microbial communities can exit through apical and lateral pathways, triggering periapical inflammation, bone resorption, and the development of apical periodontitis [59,60].

Importantly, these pathological processes are not confined to the periapical region. Anatomical pathways, including the lateral canals, furcation canals, and dentinal tubules, allow inflammatory mediators and microbial byproducts to extend toward the periodontium, giving rise to lesions that can clinically mimic primary periodontal disease [61,62,63]. In this context, cariology acts as the initiating biological trigger for a cascade that ultimately compromises periodontal attachment.

At the population level, the high prevalence of untreated or recurrent caries helps explain the frequent coexistence of endodontic and periodontal pathology in adult patients. Failure to control cariogenic biofilms at early stages, therefore, contributes not only to pulpal disease but also to complex endo–perio lesions that complicate diagnosis, treatment planning, and prognosis.

### 2.6. Clinical and Preventive Implications

Recognizing cariology as the initiating event in the tooth–pulp–periodontium continuum has important clinical implications and fundamentally reshapes decision-making across restorative, endodontic, and periodontal disciplines. Early control of lesion activity, conservative operative strategies, and durable coronal sealing can reduce toxin diffusion toward the pulp and may prevent progression to persistent pulpitis, pulpal necrosis, and downstream apical or endo–perio disease. This continuum-based perspective highlights that choices made during caries management can directly influence whether the pulp remains within a reversible inflammatory range or progresses toward necrosis and infection, setting the stage for pulpitis as the next critical biological crossroads in disease progression [64,65]. Taken together, these data support a continuum-based model in which cariology acts as the initiating event for downstream pulpal, periapical, and periodontal pathology [49,66,67]. The major biological stages of this continuum and their key clinical implications are summarized in Table 1.

## 3. Pulpitis: The Central Biological Crossroad

### 3.1. Pulpal Inflammation: A Spectrum Rather than a Binary Diagnosis

Pulpitis represents an intermediate stage in the continuum from dental caries to more advanced endodontic and periodontal disease; therefore, it is critical to determine whether a tooth will remain vital or will undergo further deterioration to the level of pulpal necrosis/endodontic infection and then to higher levels of both diagnostic and therapeutic difficulty [72,73] (Figure 1). Clinical guidelines have shifted away from treating “reversible” and “irreversible” pulpitis as strict histologic entities and instead use these clinical diagnostic categories primarily to guide treatment decisions [74]. Therefore, the distinction between reversible and irreversible pulpitis may not correspond to specific histological characteristics of the pulp. For example, a patient with deep caries or extensive restorations or altered sensory responses may not experience the amount of pain that would correlate with the degree of pulpal inflammation present [74,75]. Biologically, the pulp is a soft tissue within a low-compliance chamber surrounded by rigid dentin. Therefore, even small amounts of increased tissue pressure or vascular congestion can result in significant pain. Additionally, a pulp that has undergone significant inflammation may at times show a diminished or absent pain response due to reduced neural function within the inflamed pulp [73,75,76]. Progression of the carious process promotes diffusion of acidic, antigenic, and bacterial byproducts through dentin and dentinal tubules toward the pulp. These substances irritate the pulp, stimulate neurogenic inflammation, and activate immune responses [77,78]. Neuropeptides released from pulpal nerves, along with local mediators (i.e., prostaglandins, cytokines), stimulate vasodilation and attract immune cells. If the stimulus causing the initial irritation is removed and the lesion is properly sealed, the body’s response to the stimulus may be limited to the area of the irritation and reversible [79,80].

A major limitation is that most chairside tests assess sensibility (neural response) rather than true pulpal vitality (blood supply). While cold testing and electric pulp testing provide useful information, no single test can definitively confirm the histologic status of pulpitis; the results should be interpreted in combination with clinical and radiographic findings [81,82]. Therefore, the clinician must interpret the results of these tests in conjunction with the depth and activity of the caries, the patient’s restorative history (which includes the risk of microleakage), and any radiographic evidence of periapical lesions and periodontal probing patterns. Modern diagnostic framework approaches include the use of structured assessments and repeated evaluations rather than relying on single findings to guide the selection of either a conservative or definitive treatment plan [76,82]. These diagnostic limitations underscore why microbial burden, lesion activity, and host factors should be considered together when predicting pulpal fate and selecting conservative versus definitive management.

Although clinical diagnostic terms such as reversible and irreversible pulpitis guide treatment, their correlation with histologic pulpal status is imperfect. Symptoms may not reliably reflect inflammation, and sensibility tests assess neural response rather than true vascular vitality. Diagnostic accuracy also varies across studies, populations, and protocols. Therefore, symptom-based conclusions should be interpreted cautiously, with structured reassessment essential, especially in deep caries and heavily restored teeth [13,81,82].

### 3.2. Microbial Burden, Lesion Activity, and Host Response

Whether a tooth develops persistent pulpitis as a result of deep caries is not solely dependent upon the depth of the caries. Rather, it is the product of an interplay between microbial factors (i.e., biofilm load, virulence, metabolic activity) and the host response (vascular, immune, reparative capabilities) [75]. If the caries are highly active and produce large amounts of acid that continuously diffuse through the dentinal tubules, prolonged irritation to the pulp can occur. On the other hand, a caries lesion that is less active and thus produces less acid may give the pulp more time to react to the insult by forming tertiary dentin and containing the injury by establishing an immune response [75,83,84].

In response to the insult caused by bacterial invasion of the dentin, the pulp attempts to defend itself through innate immune mechanisms that recognize pathogen-associated molecular patterns, recruit neutrophils/macrophages, and activate adaptive immunity when inflammation persists [85]. In addition, the pulp often tries to contain the injury through the formation of reactionary/reparative dentin and limiting the penetration of bacteria through the sclerosis of dentinal tubules. However, the effectiveness of these defense mechanisms is dependent upon the duration and severity of the microbial insult and the quality of the coronal seal after operative intervention [85,86]. Systemic factors can alter the host’s ability to resolve inflammation and repair injured tissues. Examples of systemic factors that can impact the host response to a microbial insult include diabetes mellitus, smoking, age-related changes in the vasculature/cellularity, and immune modulation [87]. Clinicians should not let systemic factors alter their local findings; however, they are relevant when deciding on conservative treatments (such as vital pulp therapies) and when educating patients about the likelihood of treatment success and the need to maintain a healthy environment through proper oral hygiene and restoration maintenance.

### 3.3. Clinical Decision-Making in Deep Caries: From Conservative Caries Removal to Vital Pulp Therapy

The clinical decision-making process in deep caries management continues to be influenced by minimally invasive approaches. Selective caries removal and immediate effective sealing have been implemented to reduce the microbial load while minimizing unnecessary pulp exposure [88]. Additionally, this philosophy supports current consensus; in many instances, preserving pulp vitality promotes long-term tooth survival by allowing proper proprioception, limiting structural compromise, and reducing the risk of endodontically related complications [89].

Modern vital pulp therapy (VPT) has gained increasing acceptance and support based on evidence as an alternative treatment option in select cases of deep caries with symptoms of pulpitis [90]. VPT encompasses various procedures, including indirect pulp treatment, direct pulp capping, partial pulpotomy, and full pulpotomy [91]. Vital pulp therapy is based on biological principles, where the removal of inflamed pulp tissue to a level that allows for hemostasis and infection control, followed by the placement of bioactive materials and a proper seal, supports the healing and preservation of pulp vitality [90]. Current papers emphasize the importance of selecting patients appropriately, employing strict aseptic techniques, achieving hemostasis, utilizing appropriate biomaterials (particularly hydraulic calcium silicate-based products), and restoring the tooth immediately and effectively to achieve successful results [92,93].

Communication regarding what VPT represents and what it does not is also paramount. VPT does not eliminate the need for root canal therapy in all symptomatic teeth. Instead, it provides a conservative intervention and interceptive pathway, particularly when the tooth is strategically important, restorable, and allows the implementation of strict protocols [90,93]. Within the framework of a continuum of care, these interceptive points are fundamental. They provide the opportunity to intervene early and prevent the progression of pulpal necrosis, apical periodontitis, and endo–perio overlap, which contributes to complicated periodontal outcomes.

Although evidence supporting vital pulp therapy (VPT) has grown, reported success rates remain variable. This variation reflects differences in diagnostic criteria for irreversible pulpitis, biomaterials, restorative protocols, hemostasis methods, quality of definitive coronal restoration, and follow-up duration. Many studies are also performed in specialist or academic settings, limiting generalizability to routine practice. Therefore, VPT outcomes should be interpreted cautiously. VPT should be presented as a tooth-preserving option for carefully selected cases, not as a universal replacement for root canal therapy. Further high-quality comparative studies using standardized diagnostic criteria, long follow-up, and patient risk profiles are needed to clarify which cases benefit most [90,91,92,93].

### 3.4. Pulpal Necrosis: Gateway to Endodontic Infection and Periradicular/Periodontal Manifestations

Pulpal necrosis creates a new biological environment that promotes endodontic infection and periradicular manifestations. The root canal environment functions as a relatively isolated ecological niche that provides protection to microorganisms from the host’s defense mechanisms and allows them to proliferate, form biofilms, and resist clearance by the immune system [94,95]. At this time, the clinical objectives shift from managing inflammation to managing endodontic infection and preventing reinfection due to coronal leakage. The host response is now focused primarily at the periradicular level, where inflammatory mediators induce bone resorption and result in apical periodontitis [96,97].

This transition point is pivotal to interdisciplinary care since endodontic infection can produce manifestations similar to those of periodontal disease [86,98]. Drainage tracts may extend coronally along the periodontal ligament space and cause localized deep probing defects, furcation involvement through accessory canals, or a sinus tract opening in the sulcus. As clinicians focus primarily on probing depth, these manifestations can be mistaken for primary periodontal destruction. Conversely, true periodontal disease may exist simultaneously and ultimately lead to secondary endodontic involvement, further emphasizing the need for a systematic differential diagnosis [99,100].

Additionally, the importance of the coronal seal increases once necrosis has occurred. Although endodontic therapy may be performed in a technically proficient manner, therapy can fail if the canal becomes contaminated through microleakage or failure of the restoration [101]. Thus, restorative planning is not a follow-up procedure but rather an integral component of biologically sound endodontic–periodontal treatment [102]. The continuum approach illustrates the interconnected nature of these components: caries initiation, pulpal inflammation, infection ecology, restoration quality, and periodontal health are mutually dependent. Because endodontic infection following pulpal necrosis can mimic periodontal breakdown through shared anatomical pathways, accurate classification and structured diagnosis become essential to avoid mismanagement [103,104].

## 4. Endodontics as the Clinical and Biological Interface

### 4.1. Apical Periodontitis: Extension of Endodontic Infection

#### 4.1.1. Pathogenesis of Apical Periodontitis

Apical periodontitis (AP) is a host-driven inflammatory reaction that occurs in response to microbial invasion of the root canal system. It is associated mainly with anaerobic, polymicrobial biofilms that colonize necrotic pulp tissue and release virulence factors capable of spreading beyond the apical foramen [105]. Microbial products activate immune cells, including macrophages and dendritic cells, which then trigger a cycle of cytokine-induced inflammation. This process is characterized predominantly by interleukin-1β (IL-1β), tumor necrosis factor-α (TNF-α), and prostaglandin E_2_ (PGE_2_), leading to the degradation of periradicular tissue [106]. Persistent intraradicular infection continues to stimulate antigen production, thereby impeding resolution and leading to the development of granulomatous tissue [107].

#### 4.1.2. Host–Microbe Interactions Beyond the Apex

Microbial metabolites and structural components, such as lipopolysaccharides (LPSs) and lipoteichoic acid (LTA), are released through apical constriction, activating receptors on immune cells within the periradicular tissue and thereby amplifying periradicular inflammation [108]. In the acute phase, neutrophils are predominant and release reactive oxygen species and matrix metalloproteinases (MMPs). Conversely, chronic lesions are characterized by responses primarily involving lymphocytes and macrophages [109]. The size and progression of lesions depend on the balance between microbial virulence and the host immune response [110].

#### 4.1.3. Bone Resorption and Inflammatory Mediators

The process of periradicular bone resorption is mediated by the activation of osteoclasts through the receptor activator of nuclear factor κB ligand (RANKL) pathway. This activation is modulated in response to inflammatory cytokines produced within the lesion [111]. The severity and persistence of the lesion depend upon elevated RANKL/OPG ratios [112]. In addition, a synergistic environment is created for bone loss by macrophage-derived TNF-α and IL-6, which increase osteoclastogenesis, whereas bacterial lipopolysaccharide (LPS) directly stimulates osteoclast precursors [113]. Clinically successful endodontic treatment reduces the effect of these mediators and supports osteoblastic repair, leading to healing, which is visible radiographically [114]. These pathways elucidate the mechanisms by which persistent intraradicular infection can sustain periradicular inflammation and, in specific anatomical contexts, affect periodontal outcomes through shared communication routes.

### 4.2. Endodontic Factors Influencing Periodontal Outcomes

#### 4.2.1. Coronal Leakage and Reinfection

The long-term effectiveness of endodontic treatment is largely influenced by coronal leakage. This leakage permits bacteria from saliva to re-enter the root canal system, potentially causing inflammation to increase again in the apical region [115]. The onset of reinfection can result in chronic apical periodontitis (AP). This can lead to aggravation of periodontal breakdown when microbial byproducts pass through anatomical communication pathways [116]. When executed correctly, coronal restorations can mitigate the reinfection risk and contribute to periodontal stability by creating a sealed environment [117].

#### 4.2.2. Missed Canals and Complex Anatomy

Root canal therapy is intended to achieve a complete seal of the anatomical spaces within the canal system; however, this outcome is seldom achieved with absolute precision. Anatomical spaces such as isthmuses, fins, and accessory canals allow the colonization of remaining biofilms, which can lead to chronic inflammation and affect periodontal tissues through communication in the lateral or apical areas [118]. In root canal treatment, canals, particularly molars and incisors, can be overlooked, which may result in apical periodontitis (AP) and periodontal issues [119]. The identification of intricate anatomical structures can be enhanced by the use of cone beam computed tomography (CBCT), potentially leading to better endodontic and periodontal outcomes [120].

#### 4.2.3. Iatrogenic Factors (Perforations, Over-Instrumentation, Vertical Root Fractures)

Errors occurring during root canal procedures can result in perforations that permit microbial infiltration into periodontal tissues. If these perforations are not promptly sealed, they may lead to inflammation and subsequent attachment loss [121]. Over-instrumentation can lead to the extrusion of bacteria and debris, worsening inflammation in the peri-radicular area, and delaying healing [122]. Vertical root fractures (VRFs) are characterized by narrow and deep periodontal pockets, which can result in bone loss due to bacterial invasion through fracture lines, often necessitating extraction [123]. Collectively, these factors show how persistent or recurrent endodontic infection and iatrogenic pathways can shape periodontal signs, prognosis, and sequencing decisions in endo–perio lesions.

### 4.3. Endodontic Treatment as a Modulator of Periodontal Healing

#### 4.3.1. Resolution of Sinus Tracts and Pseudo-Pockets

Infections originating from endodontic sources may manifest as sinus tracts or periodontal pockets due to the localized degradation of the periodontal apparatus. Successful root canal treatment effectively eradicates the source of infection, which leads to the resolution of sinus tracts and the reattachment of pseudo-pockets without the need for periodontal therapy [124]. This underscores the importance of distinguishing between lesions of endodontic origin and those of periodontal origin.

#### 4.3.2. Impact of Successful Root Canal Therapy on Attachment Level

Root canal therapy involves disinfection of the root canal system, followed by its obturation. This process significantly diminishes the inflammatory mediators responsible for attachment loss in the periodontium. The literature supports improvements in clinical attachment levels and probing depths after root canal treatment [97]. The healing process is significantly better if the periodontal tissues are not affected by intraradicular infection.

#### 4.3.3. Endodontic First Approach: Biological Rationale

Prioritizing the treatment of infections of endodontic origin before addressing periodontal issues is logical, as these infections are the primary cause of inflammation in combined endodontic–periodontal lesions [125]. When inflammation originating from the endodontic area is addressed by lowering the microbial presence and inflammatory mediators, periodontal healing and eventual regeneration are promoted in a conducive environment [126]. This method also helps prevent the contamination of periodontal tissues by pathogens originating from the root canal system. In conclusion, apical periodontitis is indicative of host-mediated inflammation induced by intraradicular infection. Effective endodontic therapy reduces the microbial burden and inflammatory mediators, creating conditions that may support periodontal stability and healing. These concepts provide a biological rationale for prioritizing the management of endodontic infection in lesions of endodontic origin.

## 5. Endodontic–Periodontal Interrelationships: Biological Pathways

### 5.1. Anatomical Communication Between Pulp and the Periodontium

#### 5.1.1. Apical Foramen

The apical foramen serves as the principal channel for communication between the pulp and the periodontium, allowing for the unimpeded transfer of microbial toxins and inflammatory mediators from the necrotic pulp [127]. When pulpal necrosis and microbial colonization occur, this opening serves as the main pathway for bacterial toxins, metabolic byproducts, and inflammatory mediators to enter periapical tissues [128]. The permeability of this region facilitates the entry of immune cells from periapical tissues into the canal space; however, their defensive capabilities are limited by the challenging intraradicular environment. The dissemination of inflammation, as well as the movement of microbes and their byproducts, is contingent upon the size and structural characteristics of the apical foramen.

#### 5.1.2. Lateral and Accessory Canals

Microbes and their byproducts are exchanged in the upper third of the root and the furcation area via the lateral and accessory canals [129]. The lateral and accessory canals serve as secondary pathways connecting the pulp and periodontium. These channels extend from the primary root canal system and are predominantly located in the coronal and middle root sections, as well as in the furcation areas of multirooted teeth. When the pulp becomes infected, bacteria and byproducts travel through these canals, causing periodontal inflammation even without primary periodontal disease [127]. The intricate architecture of the lateral and accessory canals poses significant challenges to achieving effective disinfection during root canal procedures.

Periodontal issues arising from infected lateral canals can be effectively addressed through root canal therapy. The combination of thorough chemomechanical debridement and the use of irrigants capable of penetrating complex anatomical structures significantly reduces the microbial presence within the canal system [130]. Following the successful eradication of the infection within the root canal and the subsequent adequate filling of the canal, there is a reduction in the inflammatory signals transmitted through the lateral canals.

#### 5.1.3. Furcation Canals

Accessory canals located in the furcation region are prominent in molars, and their infection may manifest as originating from periodontal sources [131]. These auxiliary pathways, located in the interradicular region of multirooted teeth, serve as essential anatomical connections between the pulp and the periodontium. When the pulp becomes necrotic and infected, microorganisms and their byproducts can travel through these furcation canals, leading to localized periodontal inflammation that may appear similar to primary periodontal disease in a clinical setting [132]. Diagnosis and treatment planning may become complicated because of the extension of inflammation into the interradicular area. This can complicate the assessment of the size of the lesion by obscuring the radiographic landmarks. A detailed radiographic and clinical assessment is paramount for differentiating between inflammatory and noninflammatory defects. Treatment planning should involve advanced radiographic methods and account for the complex anatomy needed for effective management.

#### 5.1.4. Dentinal Tubules and Cement Defects

Within the root structure, DTs constitute a natural microscopic network extending from the pulp chamber to the outer surface of the root. Under healthy conditions, these tubules are protected by an intact layer of cementum, which functions as a barrier to prevent microbial intrusion. The exposure of dentinal tubules may occur as a result of periodontal infection or therapeutic interventions such as root planing, which may allow for the penetration of bacteria and communication between the pulp and the periodontal tissues [133]. Defects within the cementum increase the permeability and allow toxins from microbes to traverse the root surfaces. Microbial toxins and metabolic byproducts can easily pass through exposed root surfaces, reaching the periodontal ligament or penetrating further into the dentinal tubules [134]. The bidirectional movement of irritants can exacerbate both pulpal and periodontal inflammation, thereby complicating diagnosis and treatment planning. These communication pathways elucidate how diseases originating in the pulp can clinically manifest within periodontal tissues, underscoring the necessity of pathway-based classification for precise diagnosis and treatment sequencing.

### 5.2. Classification of Endo–Perio Lesions Revisited

#### 5.2.1. Primary Endodontic Lesions

Primary endodontic lesions originate from an infection within the pulp, which progresses toward the root apex and subsequently ascends through the periodontal ligament [135]. As the pulp tissue dies, microbial toxins, enzymes, and inflammatory substances escape through the apical foramen and accessory pathways, leading to localized periodontal damage. These lesions originate within the pulp and may drain coronally through the periodontal ligament, commonly presenting as narrow pockets that heal after root canal treatment [136].

#### 5.2.2. Primary Periodontal Lesions

Primary periodontal lesions originate within periodontal tissues and progress toward the root, as plaque-induced inflammation compromises the tooth’s supporting structures [135]. As periodontal disease progresses, the progressive loss of attachment and bone may expose the lateral and accessory canals, thereby establishing new connections between the periodontium and the pulp. As periodontal deterioration extends apically, it may uncover accessory canals and, in severe cases, jeopardize pulpal vitality [137]. Periodontal therapy is employed to address these lesions, whereas endodontic treatment becomes necessary solely in instances where the pulp undergoes necrosis.

#### 5.2.3. True Combined Lesions

True combined lesions are characterized by the convergence of distinct endodontic and periodontal pathologies, progressing to a point where they coalesce into a singular, continuous defect impacting both the pulp and periodontal structures [135]. When combined, endodontic and periodontal lesions can lead to significant defects that necessitate both endodontic and periodontal interventions [138]. The outcome of these lesions is contingent upon the extent of periodontal damage. While root canal treatment can typically address the endodontic component with a high degree of predictability, the periodontal component may require regenerative techniques, surgical interventions, or ongoing maintenance therapy [139].

#### 5.2.4. Iatrogenic and Trauma-Related Lesions

Defects caused by medical interventions or trauma, such as external or internal resorption, vertical or horizontal fractures, and root perforations, can unintentionally create channels of communication between the pulp and periodontal tissues [140]. The alteration of these structural components compromises the inherent protective barriers of the root surface, thereby facilitating the unrestricted movement of microorganisms, inflammatory agents, and toxins between these two regions. The treatment of lesions caused by medical procedures or trauma often requires a combination of endodontic and periodontal strategies [103]. Endodontic therapy is essential for the eradication of infections within the root canal, whereas periodontal treatment may be required to address external damage and facilitate tissue regeneration [141]. In summary, this classification highlights the critical importance of identifying the primary source and pathway of dissemination for determining prognosis and establishing the sequence of treatment in endo–perio lesions.

### 5.3. Microbial Similarities and Cross-Contamination

#### 5.3.1. Shared Anaerobic Microbiota

An important aspect of the interaction between endodontic and periodontal conditions is the presence of a common anaerobic microbiota in both diseases, such as *Porphyromonas gingivalis*, *Fusobacterium nucleatum*, and *Prevotella intermedia*, which are often found in infections originating from both the pulp and periodontal tissues [142]. Their presence in these distinct yet interconnected regions highlights the potential for microbial transfer between the endodontium and periodontium [134].

#### 5.3.2. Biofilm Resilience Across Tissues

Biofilms are present in niches of the periodontal and endodontic areas [143]. These complexly organized microbial communities adhere to surfaces in both periodontal and endodontic environments. These bacteria exhibit resistance to antimicrobial agents due to the presence of extracellular polymeric substances and synergistic microbial interactions [144]. The combination of low oxygen levels, nutrient gradients, intricate anatomical structures such as dentinal tubules and lateral canals, and irregularities on root surfaces creates optimal conditions for biofilms to thrive [130]. Within these biofilms, bacteria can engage in quorum sensing, allowing them to coordinate their actions and adjust to environmental challenges, thereby increasing their durability. Persistent biofilms can sustain chronic inflammation and complicate treatment [145].

#### 5.3.3. Antibiotic and Host Response Implications

The management of endo–perio lesions is influenced by both the microbial composition of the infection and the host’s immune response [146]. The selection of antibiotics is affected by the profiles of the microbes and emphasizes the importance of host-modulating therapies [147]. Antibiotics alone often prove inadequate because biofilms offer protection, and necrotic pulpal tissues have a limited blood supply [148]. This underscores the importance of host-modulating therapies, which focus on controlling the harmful aspects of the immune response instead of merely targeting microorganisms.

Under both endodontic and periodontal conditions, inadequate regulation of the host’s immune response hastens the destruction of tissues. Increased levels of proinflammatory cytokines such as IL-1β, TNF-α, and IL-6, along with matrix metalloproteinases (MMPs), play a role in the breakdown of collagen and alveolar bone [149]. These mediators, which are elevated during chronic infections, sustain a cycle of inflammation and tissue damage. When the host’s response becomes imbalanced, even minor microbial challenges can result in excessive periodontal damage.

The recognition of these immunological dynamics has led to the incorporation of host-modulating strategies into treatment protocols. In instances of combined endo–perio lesions, the management of the host response is essential, as inflammation originating in one tissue area can exacerbate damage in another [150]. By combining antimicrobial approaches with host modulation, healthcare providers can foster a more conducive environment for healing and enhance long-term treatment results [151].

There is an intricate relationship between the pulp and the periodontium. As previously discussed, this connection is facilitated through various pathways that enable the transmission of disease from one to the other. These pathways shape the clinical presentation of different types of endodontic–periodontal lesions. Hence, it is extremely important to classify and diagnose these lesions to perform accurate treatment and sequencing. It is also clear that both these tissues share anaerobic microbes and resilient biofilms, which can lead to persistent infection and difficulties in management. The exacerbation of tissue damage can occur due to a dysregulated response from the host tissue. Collectively, these shared microbiological and host response characteristics elucidate why lesions of endodontic origin can resemble periodontal breakdown and why the management of combined lesions often necessitates a staged interdisciplinary approach.

## 6. Periodontal Breakdowns Influenced by Caries and Pulpal Disease

### 6.1. Periodontal-like Breakdown Secondary to Endodontic Pathology

A key challenge in the assessment of caries–pulp–endo–perio mechanisms is that endodontic disease can present with clinical manifestations similar to those of periodontal disease. These include isolated deep probing defects, swelling, suppuration, and sinus tracts draining through the gingival sulcus [103,152]. As a result, findings that appear consistent with marginal plaque-induced periodontitis may, in selected cases, originate from intraradicular infection [152]. Periodontally speaking, the most common clinical error is the assumption that all deep probing depths represent periodontal pockets. It is possible that a small, isolated defect on one surface of a single tooth in a patient without a general pattern of periodontitis may represent a drainage pathway from an endodontic source [104]. Likewise, furcation involvement can occur through accessory canals and anatomical communications; the presence of a furcation finding does not necessarily indicate that the condition was caused by marginal periodontitis [153,154]. As such, this pattern illustrates the importance of interpreting lesion morphology (whether the lesion is narrow or broad), the distribution of the lesion (whether it is localized or generalized), and other findings (such as the presence of a sinus tract, vitality of the involved tooth(s), and any radiographic evidence of periapical disease) [154,155].

Endodontic-origin periodontal-like defects have been reported to significantly improve once the infection has been controlled and the coronal portion of the root canal system has been sealed [156]. Once the intraradicular reservoir of infection has been eliminated, drainage stops, inflammation decreases, and the probing depth can decrease significantly, sometimes returning to near-normal levels [154]. In contrast, treating periodontal disease alone will be unsuccessful until canal infection has been eliminated, since the primary source of inflammation has not been removed. Therefore, the best approach is to provide a two-stage interdisciplinary plan: treat suspected endodontic infection first (if necessary), assess the periodontal health of the teeth after the initial stages of healing, and then proceed with the treatment of the remaining periodontal disease [104,152].

### 6.2. Endo–Perio Lesions as a Continuum: Why Boundaries Blur

The ambiguity between endodontic and periodontal disease is not merely a semantic issue but also a reflection of the real biological and anatomical communication between the two systems [103]. Communication pathways exist between the two systems, including the apical foramen, lateral/accessory canals, furcation canals, and dentinal tubules (especially when exposed), and iatrogenic or structural pathways such as cracks and perforations. Through these communication pathways, microbial products and inflammatory cells can migrate from one compartment to another, creating the clinical phenomenon referred to as endo–perio lesions [104,152]. A further complicating feature of the misclassification of disease is that a disease process may be viewed solely by the discipline responsible for its management. For example, a periodontist may evaluate a patient’s probing depth without considering the patient’s vitality or the presence of a sinus tract. Conversely, an endodontist may attribute signs of infection to canal infection without evaluating the patient’s generalized periodontal status. Furthermore, combined lesions present additional challenges to decision-making, as both marginal plaque-driven disease and endodontic infection may be present, each providing a continued source of inflammation and impeding healing [98,103,157]. Therefore, a useful framework to consider lesions is based upon their origin and pathway rather than solely on their appearance [103,104,155].

The relevant clinical questions to ask are as follows: Where did the problem originate? Is there evidence of persistent intraradicular infection? Is periodontal attachment loss generalized or localized? Are there anatomical features (furcation canals, lateral canals) or acquired defects (cracks) that explain the clinical presentation? The answers to these questions will directly inform the sequence of treatments to be provided as well as the prognosis for the patient. This pathway-based reasoning provides the foundation for the structured diagnostic workflow presented in Section 6.3.

### 6.3. Root Surface Caries, Cervical Lesions, and Restoration Margins as Periodontal Risk Modifiers

Endodontic pathology is only one of several pathways through which changes from caries can affect periodontal health. The local ecological and structural effects of caries-related alterations on the root surface can also cause periodontal instability. When gingival recession occurs, there is a high likelihood that the exposed root will experience caries (root caries). These types of lesions have the potential to cause problems such as increased plaque retention, difficulty in cleaning, and chronic inflammation of the gingiva. The same principles apply to cervical lesions, whether they are caused by caries or other factors. These lesions provide areas for biofilm accumulation and can create favorable conditions for biofilm stagnation, especially if the marginal area of the restoration is rough, overcontoured, or placed subgingivally. Thus, the clinician must consider the location and contour of the restoration margins in addition to the overall condition of the periodontium. If the restoration invades the biological width zone or creates areas of plaque stagnation, then it can lead to chronic inflammation and eventual attachment loss [158].

The number of times a tooth requires restoration may also increase the risk of microleakage, which in turn can increase pulpal stress and potentially accelerate the progression toward pulpitis and necrosis in susceptible teeth. Therefore, this represents one of the most direct connections between cariology/restorative decision-making and periodontal outcomes within the continuum model [159,160]. Importantly, the connection between caries and periodontal disease is bidirectional. Attachment loss and recession of the periodontium increase the exposure of roots, thereby creating a greater risk of caries; conversely, root caries and cervical defects increase plaque retention and increase the difficulty of achieving good periodontal health [161]. Therefore, the management of root caries is not only a restorative issue but also a part of periodontal risk assessment and long-term maintenance planning.

### 6.4. Effects of Periodontal Therapy on Pulp-Related Signs and the Risk of Diagnostic Confusion

Periodontal therapy can impact the pulp indirectly. For example, scaling and root planing can increase the sensitivity of dentin, and surgical procedures can produce temporary alterations in local tissue conditions that can change symptom patterns [162]. In a tooth that is already damaged from a deep restoration, cracks, or experiences borderline pulpitis, these changes can reveal what appears to be a “pulpal” symptom, even though the original problem may still be related to periodontal disease or vice versa [162,163].

Therefore, the clinical concern is that symptom changes that occur following periodontal therapy can be misinterpreted as evidence of new endodontic disease; similarly, unresolved symptoms and continued localized defects following periodontal therapy can represent an untreated endodontic problem [163]. The best way to address overlapping symptoms is to use a structured approach to reassess the patient’s symptoms and condition: recheck vitality and sensibility, reevaluate probing patterns, reexamine radiographs, and consider sinus tract tracing or CBCT imaging as needed. From an interdisciplinary perspective, all periodontal treatments should be performed while considering the patient’s pulpal status and the integrity of the restorations present. Teeth with significant histories of caries, extensive restorations, or recurrent episodes of pain may require closer monitoring [164].

The continuum framework promotes the idea of thinking about the tooth as a single biological unit: the health of the pulp, the integrity of the restorative seal, the health of the root surface, the ecology of plaque, and the attachment of the periodontium are all connected in their ability to determine the outcome of the patient’s treatment. Since endodontic drainage, root surface caries, and restorative factors can complicate the interpretation of findings from periodontal assessments, these overlapping presentations highlight why diagnostic errors are common and why a structured differential-diagnosis workflow is essential to prevent inappropriate sequencing of care.

## 7. Diagnostic Challenges Across Disciplines

### 7.1. Why Endodontic and Periodontal Diseases Mimic Each Other

Clinical issues in the caries–endo–perio continuum occur because diseases from different sources can produce clinically similar findings [81] (Figure 1). For example, isolated deep probing, suppuration, swelling, and sinus tract formation may be observed in both periodontitis and endodontic infections. Similarly, periodontal disease can cause symptoms such as pain during chewing or tooth sensitivity, which may be misattributed to a pulpal problem. In addition, chronic drainage from early apical periodontitis can reduce pain and mask the endodontic origin [98,103]. Misclassification is often driven by limitations inherent to testing (i.e., sensitivity does not equal vitality), two-dimensional radiographs, and distortion of probing patterns caused by drainage tracts [103,104]. Cognitive errors also contribute, including “snapshot bias” (overcommitment to findings from a single visit) and “silo bias” (deep pockets = perio; radiolucencies = endo), without considering the broader clinical context [165]. Finally, true combined lesions can occur, and plaque-mediated periodontal disease may coexist with endodontic infection, which reinforces the need for structured assessment and planned reassessment.

### 7.2. Stepwise Differential Diagnosis Workflow (Practical Algorithm)

By incorporating all the information from Steps 1 through 3 above, a stepwise approach to differential diagnosis can reduce misdiagnosis by integrating pulp, periapical, periodontal, and restorative findings rather than evaluating each component in isolation [166,167,168,169].

Step 1—Patient medical and dental history: Obtain a detailed pain history (including sensitivity to heat/cold, duration, and whether pain is spontaneous or provoked) and document swelling episodes, analgesic response, and the timeframe of symptoms. The patient’s dental history, including their restoration history (deep restorations, multiple restorations over time, recent restoration changes), and relevant medical history, including trauma/damage to the tooth, history of cracks/fracture suspicion, and prior periodontal diagnosis or treatment, is obtained [169].

Step 2—Examination of the oral cavity: Examine for intraoral and extraoral swelling and evaluate for the presence of sinus tract opening(s) (both intraorally and extraorally). Any tenderness to palpation should be documented, and regional lymphadenopathy should be assessed when indicated. Evaluate occlusion and signs of parafunctional habits such as clenching/grinding, as these may contribute to cracks or occlusal trauma that can mimic or complicate an endodontic–periodontal presentation [169].

Step 3—Assessment of Tooth Pulp Functionality: Test pulp function via cold testing and electric pulp testing. These methods primarily evaluate the neural response rather than the pulpal blood supply; therefore, the results should be interpreted in context. The responses of adjacent and contralateral teeth were compared. A prolonged response suggests an inflammatory condition; however, it does not confirm the histological extent of inflammation. If there is no response, this may indicate pulpal necrosis or calcification. Repeat testing if the results are inconsistent with the overall clinical presentation, and correlate findings with restorability, radiographic features, and the pattern of periodontal involvement [81,82].

### 7.3. Role of CBCT: Indications, Value, and Limits

The use of CBCT to clarify ambiguity in diagnosis can improve clinical decision-making in selected cases. CBCT allows clinicians to assess lesions in three dimensions, which helps them visualize the extent of the lesion and cortical bone involvement. Additionally, CBCT can be used to assess the resorption pattern around the lesion and to look for signs of a vertical root fracture. However, CBCT is not intended to be a screening tool. Instead, it is best used when there is a high degree of certainty that the images will change the course of treatment. Interdisciplinary teams can benefit from the use of CBCT to (i) assist in clarifying the origin of an unknown lesion, (ii) assess furcation involvement patterns and possible pathways, (iii) assess the size of the lesion and its spatial relationship to adjacent structures for discussion of prognosis, and (iv) assess the morphology of the roots that could affect the feasibility of endodontic therapy. However, the information gathered from CBCT scans must always be correlated with that from clinical testing to assess the activity and etiology accurately [170,171]. Although CBCT can improve visualization of lesion extent and anatomical complexity, its diagnostic yield depends on case selection and image interpretation, and it cannot reliably distinguish active versus inactive disease without correlation with clinical tests. Therefore, CBCT should be framed as an adjunct that may change management in selected cases rather than a routine diagnostic substitute [120,171].

### 7.4. Emerging Adjuncts (Biomarkers/AI): Useful to Mention, but Evidence Still Evolving

As technology advances, biomarkers and artificial intelligence-assisted imaging interpretation are two new technologies that are being researched to further enhance the classification of disease activity and diagnostic accuracy. Biomarkers and AI will likely become useful adjuncts in the future and are relevant to mention in this section. However, at this time, they have yet to be validated or standardized and therefore should be discussed as potential adjuncts rather than established standards [166,172,173]. Once a diagnosis has been made on the basis of a structured differential diagnosis process and judicious use of adjunct imaging, clinicians can begin to develop a treatment plan on the basis of evidence-based treatment options and biological considerations to achieve the greatest level of tooth preservation and healing. Current studies on biomarkers and AI vary widely in terms of sampling methods, reference standards, external validation, and clinical endpoints; therefore, translation into routine chairside decision-making remains limited and should be interpreted as emerging rather than practice-changing evidence at this stage [166,172,173].

## 8. Therapeutic Implications: Toward Interdisciplinary Treatment Planning

### 8.1. Treatment Sequencing: The Core Strategy to Avoid Failure

Misclassification is a common cause of treatment failure in complex cases because it results in inappropriate sequencing of care (Figure 1). The rationale of modern interdisciplinary treatment planning is to identify and manage the primary source of infection or inflammation first, control drainage when present, and then reassess it before proceeding to additional interventions [174]. This sequential process is particularly important in endo–perio lesions, as the clinical presentation of the case may dramatically change following the initial treatment of the infection. Treatment of many ambiguous presentations, such as localized probing, sinus tracts, periapical tenderness, and radiographic periapical pathology, often supports an endo-first treatment approach because the intraradicular infectious reservoir is being targeted [175]. Following disinfection of the root canal system and placement of a definitive coronal seal, drainage will typically resolve, and the probing depth may improve, which will clarify if there are remaining components of the disease that require definitive treatment with periodontal therapy. Notably, the “endo-first” treatment approach is not rigid but is based upon suspicion of an endodontic etiology and is dependent upon the restorability of the tooth [176].

When generalized periodontitis exists, periodontal stabilization and biofilm control must occur simultaneously with endodontic therapy. This will include oral hygiene instruction and reinforcement, debridement, and other measures to reduce or eliminate major risk factors that could impede healing regardless of successful endodontic therapy [177]. Thus, interdisciplinary care is transformed into a coordinated plan rather than a competitive relationship between the different disciplines. Evidence supporting treatment sequencing, including endodontic-first management for suspected intraradicular disease, varies by lesion type, periodontal severity, and restorability. Therefore, sequencing should not be applied uniformly across all endo–perio presentations but should be individualized, guided by diagnosis, and reassessed after initial therapy to refine subsequent treatment decisions [103,104,175].

### 8.2. Early Interception: Preventing Progression to Endo–Perio Complexity

The continuum model illustrates the early intervention options available to prevent a tooth from entering a state of overlap between endodontics and periodontics. Examples of these include caries risk assessment and management, rapid control of lesion activity, and minimally invasive operative procedures. Selective caries removal, along with an adequate seal, can reduce the risk of pulp exposure and stabilize the environment surrounding the lesion, thereby reducing the likelihood of progression to severe pulpitis and necrosis [178].

Vital pulp therapy can also be considered an interceptive strategy that can preserve the tooth. Recent studies provide support for VPT in certain clinical situations, provided that the protocols are strictly followed (aseptic techniques, hemostasis, bioactive materials, and immediate high-quality restoration). This narrative review does not indicate that VPT is universally superior to conventional endodontic therapy; rather, it indicates that VPT provides additional options for conservative treatment approaches and can potentially limit the extent of progression to canal infection when appropriately utilized [90,91].

### 8.3. Coronal Seal and Restorability: Non-Negotiables Across the Continuum

Regardless of whether a tooth undergoes vital pulp therapy (VPT) or root canal treatment, the quality of the coronal seal, a determinant of long-term health, is perhaps the most critical factor across the entire continuum of treatment [179]. An inadequate coronal seal may permit microleakage, which could either create a new source for bacterial challenges or reintroduce existing bacterial challenges to undermine the healing process even after technically correct endodontic treatment has been completed. Thus, the restoration plan should be coordinated early in the sequence of treatment: the location of margins, the presence of a ferrule, the choice of materials, and the feasibility of maintaining the restoration over time [179].

The potential impact of restorability on the prognosis of a tooth is likely underestimated by many practitioners. For example, a tooth that has adequate periodontal support but is structurally compromised (has cracks, has inadequate ferrules, and has significant areas of bone undermining) may ultimately have a poor long-term prognosis regardless of the success of endodontic infection control, whereas a tooth that has a localized drainage-related defect but is otherwise well restored may have an excellent long-term prognosis once the source of endodontic infection is eliminated [180].

### 8.4. Periodontal Management After Endodontic Therapy: Timing and Reassessment

A practical recommendation in the management of patients who have undergone successful endodontic infection control is the timing of assessment for periodontal disease. Assessments should be performed before irreversible periodontal treatments [181]. The timeframe required for complete resolution of inflammation may be variable, and thus, assessing the need for further treatment after a specific interval provides the practitioner with the opportunity to assess whether the previously observed probing defects were due to drainage or persistent periodontal attachment loss. Once residual periodontal disease is identified, appropriate therapy can be initiated; this may include regenerative techniques if anatomically feasible, and the patient’s conditions support them [181]. Utilizing this phased approach will result in less overtreatment and enhance the practitioner’s ability to provide informed consent to their patients. Practitioners can inform their patients that the goal of the initial phase of treatment is to remove the primary source of infection; the goal of the subsequent phases is to address those aspects of periodontal disease that remain after the resolution of inflammation [163].

### 8.5. Prognosis and Tooth Preservation Decision-Making

Prognosis should be based on an interdisciplinary, patient-centered model incorporating multiple factors, including (i) periodontal status (amount and distribution of attachment loss, degree of furcation involvement, extent of mobility); (ii) endodontic feasibility (predictability of infection control, anatomical constraints, complexity of potential retreatment); (iii) structural integrity (cracks, presence of adequate ferrule, restorability, crown/root ratio considerations); and (iv) patient-related factors (ability to maintain plaque control, systemic risk factors, and compliance with scheduled maintenance) [98,163].

The consensus-based literature indicates that patients with combined lesions can vary widely depending upon the pathogenesis of each lesion. As a result, making accurate diagnoses and determining the correct sequence of therapies represent two critical modifiable factors that can influence the outcome of treatment. Utilizing a phased approach will allow practitioners to engage in collaborative decision-making processes with their patients regarding tooth retention/preservation; some teeth may be restored predictably through interdisciplinary care, whereas other teeth may be best managed through removal when structural or periodontal limitations are reached [182]. With the concepts of sequencing, coronal seal integrity, and prognosis established, the remainder of the manuscript will focus on practical application through standardized interdisciplinary workflows and education and how these strategies may reduce misdiagnosis and improve outcomes in routine clinical practice.

### 8.6. Clinical Translation: Practical Recommendations for Interdisciplinary Management

To translate the caries–pulp–endo–perio continuum into routine clinical practice, we propose the following pragmatic recommendations for diagnosis, sequencing, and long-term tooth preservation (Figure 1) [107,108,142,156].

(1) When to suspect endodontic contribution to a periodontal presentation
  An endodontic component should be suspected when there is an isolated deep, narrow probing defect; sinus tract drainage through the sulcus; localized swelling or suppuration inconsistent with the overall periodontal status; or a history of extensive caries or large restorations suggesting pulpal damage. These findings may mimic periodontal disease and require structured endodontic–periodontal evaluation [103,104,138,150,152].
(2) Structured diagnostic workflow: minimum dataset
  A step-by-step diagnostic process may also be helpful. The diagnostic steps are as follows: (1) pain history (type of pain; whether it is provoked or spontaneous; duration; and whether it occurs at night); (2) sensibility assessment (cold/EPT), with the understanding that sensibility does not necessarily equal vitality; (3) complete periodontal charting to determine whether gum disease is localized to specific areas versus generalized; (4) panoramic X-ray and sinus tract tracing as needed for each patient; and (5) re-examination after initial therapy when the diagnosis remains uncertain [81,82,103,104,167,169]. CBCT should be used selectively (not as a screening tool), particularly when it is likely to change management (e.g., suspected fracture, unclear lesion extent, complex anatomy, or surgical planning). CBCT findings must be correlated with clinical tests and periodontal patterning [120,171].
(3) Treatment sequencing guide: practical rule set
  When intraradicular infection is suspected, endodontic treatment and a reliable coronal seal should be established, followed by reassessment of periodontal healing before irreversible surgery. If generalized plaque-induced periodontitis coexists with localized disease, biofilm control and nonsurgical periodontal therapy can be performed concurrently to support stabilization and healing. Treatment sequencing should be individualized according to lesion distribution, severity of periodontal destruction, and tooth restorability rather than applied as a universal protocol [103,104,138,155,175].
(4) Upstream interception to prevent progression
  Caries risk control and minimally invasive strategies should be prioritized to reduce progression toward pulpal necrosis and endoperomatic complexity. Selective (incomplete) caries removal combined with effective sealing can reduce the risk of pulp exposure and support pulp preservation when appropriate case selection is performed. When strict protocols are met (asepsis, hemostasis, bioactive materials, and immediate high-quality restoration), vital pulp therapy (VPT) can be considered an interceptive, tooth-preserving option in selected cases [84,90,91,92,93].
(5) Nonnegotiables that determine long-term success
  Regardless of which procedure a tooth has undergone, VPT or root canal, an intact restorative coronal seal is required to avoid future infection and to promote the overall health of the supporting periodontium. The quality of the coronal restoration can potentially affect both early and long-term failure rates for root canal-treated teeth, which is why it is essential that all aspects of restorative planning are completed early on (such as margin design, ferrule placement, material selection, and restorative maintainability) [103,138,182].
(6) Practical “do/do not” summary
  Do: use structured diagnosis, trace sinus tracts when present, treat suspected intraradicular infection first when indicated, reassess before irreversible periodontal interventions, and secure a durable coronal seal early [107,108,120,177].
  Do not: assume that isolated deep pockets represent primary periodontitis without pulpal assessment, rely on a single test in ambiguous cases, or proceed to definitive periodontal surgery without reassessment when an endodontic source is plausible [84,85,107,108,177].
  Clinical take-home: Applying this continuum-based framework as a structured diagnostic and sequencing guide can reduce misclassification, avoid overtreatment, and improve predictable long-term tooth preservation [103,104,116,175].

## 9. Educational Implications: Aligning Training with the Biological Continuum

The interactions delineated in the preceding sections underscore the integration of caries, endodontics, periodontics, and restorative variables into a continuous spectrum, rather than as distinct, isolated domains as previously considered. This perspective is crucial from an educational standpoint in dentistry. To prevent misdiagnosis, dental students need to comprehend the shared pathways and diverse clinical presentations. This necessitates a curriculum that transcends isolated boundaries. Furthermore, both dental students and clinicians must holistically interpret clinical findings and employ a structured diagnostic approach.

### 9.1. Gaps in Undergraduate and Postgraduate Training

#### 9.1.1. Discipline-Based Barriers in Dental Education

Globally, most dental education comprises departmental divisions under which cariology, operative dentistry, periodontics, and endodontics are delivered as separate sections. This leads to “fragmented learning” for the students, even though all these subspecialities overlap in terms of microbial flora, host immune responses, and anatomical pathways [183]. Learners ultimately develop strong skill sets to manage individual specialties; however, they are unable to follow a combined management model, which is indicative of the interconnected biological reality of dental tissues.

#### 9.1.2. Lack of Integrative Case-Based Learning

The students’ clinical training followed the same segmentation process. They manage caries in operative dentistry clinics, pulp inflammation in endodontic clinics, and periodontal disease in periodontic clinics. This shows a limited ability to follow the pattern of disease from the beginning to the end. This becomes a problem, as they are unable to identify the boundaries of deep carious lesions and early pulp inflammation. Similarly, the abilities of necrotic pulp to affect periodontal tissues and advanced periodontal disease to affect pulp tissue remain challenging. Research in clinical education supports the fact that integrated case-based learning in dentistry improves the clinical diagnosis of complicated cases [184].

### 9.2. Proposal for an Integrated Disease-Based Curriculum

#### 9.2.1. Teaching Caries, Pulpitis, and Periodontal Disease as a Continuum

It is important to consider dental diseases as integrated and interconnected conditions rather than individual entities in the dental curriculum. The focus of teaching should be on caries as a biofilm-initiated and host-regulated event, followed by pulp inflammation and its progression from pulp necrosis to apical periodontitis and the involvement of the periodontium.

#### 9.2.2. Interdepartmental Clinical Teaching Models

The dental schools need to implement interdepartmental teaching strategies, which could be in the form of integrated case presentations. It is important to have comprehensive dental clinics where students can handle cases across the entire spectrum of disease presentations. Additionally, incorporating joint supervision sessions with specialists from various fields would be beneficial. This strategy can better equip dental graduates to handle real-world clinical situations more cohesively.

### 9.3. Future Directions in Curriculum Reform

#### 9.3.1. Competency Frameworks Based on Biological Pathways

Competence-based curricula should be implemented globally, with skills focused on managing biological processes, for example, “*diagnosing and managing periodontal pulp inflammation*” or “*diagnosing endodontic periodontal disease*,” rather than “*performing scaling root planning*” or “*performing root canal treatment.*” This is consistent with the contemporary approach to competency-based education [185].

#### 9.3.2. Longitudinal Patient-Centered Care Models

Dental schools should ensure clinical models in which students are allocated patients whom they can follow over a longer period. This will allow them to follow the progression of the disease and the outcome of the treatment. This will help replicate real-time clinical practice.

#### 9.3.3. Faculty Development for Interdisciplinary Teaching

Schools should incorporate training programs to prepare faculty to teach across specialties, work via an interdisciplinary approach, and collaborate during clinical supervision sessions.

### 9.4. Limitations, Diagnostic Uncertainty, and Translational Considerations

As a narrative review, this paper synthesizes evidence from animal, in vitro, clinical outcome, and observational studies that differ in quality, endpoints, and generalizability. Mechanistic findings may not reliably predict clinical outcomes. Diagnostic uncertainty is further increased by the imperfect correlation among symptoms, sensibility testing, radiographic findings, and true histologic pulpal status, since sensibility does not equal vitality. Clinical success also depends on patient selection, lesion type, operator protocols, restorative quality, and follow-up duration, limiting direct study comparisons and preventing universally applicable practice recommendations.

## 10. Conclusions

This narrative review presents an integrated biological and clinical continuum linking caries-induced microbial imbalance with dentin-mediated pulpal inflammation, pulpal necrosis, intraradicular infection, apical periodontitis, and periodontal-like breakdown through shared anatomical pathways. Clinically, this continuum explains why endodontic and periodontal diseases may appear similar, supports systematic differential diagnosis based on disease stage and severity, and guides sequential treatment planning, including an endodontic-first approach when an intraradicular source is suspected. The review also emphasizes that early intervention, through caries prevention and control, selective caries removal, and vital pulp therapy when indicated, may reduce progression toward advanced disease. Early infection control and durable coronal sealing are highlighted as critical modifiable factors influencing prognosis and long-term tooth preservation. Overall, this review integrates biological mechanisms from cariology, endodontics, and periodontology with practical clinical applications, supporting a continuum-based model for diagnosis, treatment sequencing, and interdisciplinary tooth-preserving care.

In many cases, this process begins with caries. As the lesion advances, dentin acts as a key pathway, allowing bacterial byproducts to reach the pulp. This leads to pulpitis, a critical turning point where the future course of the disease is determined. If the disease is not effectively controlled, the pulp may become necrotic, resulting in an infection within the root canal system. This infection can extend beyond the tooth through shared anatomical pathways, influencing surrounding periodontal tissues and sometimes creating overlapping clinical presentations. Understanding this continuum is essential for making accurate diagnoses, planning the correct sequence of treatment, and avoiding mismanagement, especially in complex endodontic–periodontal cases. Clinical success depends on early intervention, minimally invasive approaches, preserving pulp vitality whenever possible, controlling infection effectively, and maintaining a strong coronal seal. By adopting this biologically driven, continuum-based perspective, clinicians can foster better interdisciplinary collaboration, improve the predictability of treatment outcomes, and ultimately support long-term tooth preservation. Clinically, applying this continuum-based framework as a structured diagnostic and sequencing guide can reduce misclassification, avoid overtreatment, and improve predictable long-term tooth preservation.

## Figures and Tables

**Table 1:** Cariology as the Initiating Event in the Tooth–Pulp–Periodontium Disease Continuum.

Disease Stage	Biological Mechanisms	Clinical Consequences
Early enamel caries [23,24,25,26,27,28,29,30,31]	Biofilm dysbiosis; acidogenic/aciduric shift; ecological plaque imbalance	White spot lesions; early demineralization
Dentin caries [14,18,39,40,41,42,68]	Increased dentin permeability; diffusion of acids, LPS, and proteases through dentinal tubules	Deep lesions; early pulpal inflammation without bacterial invasion
Reversible pulpitis [43,44,45,46]	Odontoblast TLR activation; cytokine release; reactionary dentin formation	Thermal sensitivity: localized, potentially reversible inflammation
Irreversible pulpitis/necrosis [47,48,59,60,69]	Breakdown of pulp defense, sustained inflammation; microbial penetration	Spontaneous pain; loss of pulp vitality; bacterial colonization of the pulp chamber
Apical periodontitis [12,15,17,59,60,69]	Cytokine-driven osteoclastogenesis; periapical inflammation and bone resorption	Periapical radiolucency; tenderness to percussion; possible swelling
Endo–perio lesions [20,61,62,63]	Spread via lateral canals, furcation, and dentinal tubules; overlapping microbial communities	Combined periodontal attachment loss and periapical destruction
Operative modulation [49,50,51,52,53,54,55,56,57,66,67,70,71]	Selective caries removal; microleakage; material biocompatibility	Restoration-induced pulpitis; late pulpal necrosis; restoration failure
Preventive modulation [36,37,38,64,65]	Risk-based caries management; biofilm control; remineralization	Reduced incidence of pulpitis and downstream endo–perio sequelae

LPS—lipopolysaccharides; TLR—Toll-like receptor.

## Data Availability

No new data were created or analyzed in this study.

## Abbreviations

ADA	American Dental Association
AP	Apical periodontitis
CBCT	Cone beam computed tomography
EPL	Endodontic–periodontal lesion
EPT	Electric pulp test
IL	Interleukin
LPS	Lipopolysaccharide
MMPs	Matrix metalloproteinases
OPG	Osteoprotegerin
PAI	Periapical index
PGE_2_	Prostaglandin E_2_
RANKL	Receptor activator of nuclear factor κB ligand
RCT	Root canal treatment
TLR	Toll-like receptor
TNF-α	Tumor necrosis factor-alpha
VPT	Vital pulp therapy
